# Supplementary material for: Nosocomial Outbreak of SARS-CoV-2 in a “Non-COVID-19” Hospital Ward: Virus Genome Sequencing as a Key Tool to Understand Cryptic Transmission
Source: Viruses. 2021 Apr 1;13(4):604. doi: 10.3390/v13040604 (PMC8065743; doi:10.3390/v13040604)
Supplement: Supplementary file 1 [file viruses-13-00604-s001.pdf]

Supplementary Material

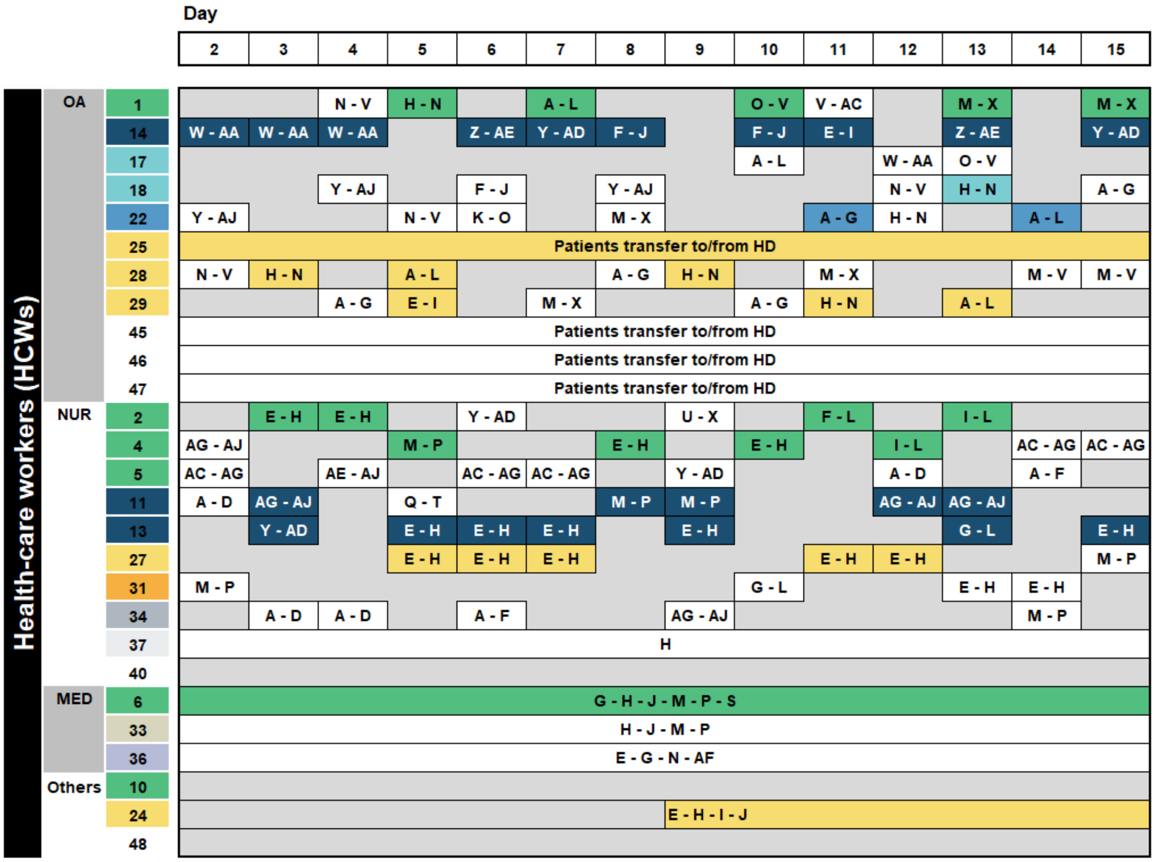

Figure S1. Timeline of HCW-patient contacts before the large screening. HCW and patient codes are colored according to the phylogenetic placement of the respective SARS-CoV-2 consensus sequences (Figure 1A), which exception of those without SARS-CoV-2 genome data (gray numbers in white fields). The timeline (matching that of Figure 1, in which day 1 corresponds to the first day of the 15-day period before the large screening) summarizes the visits of infected HCWs to patient's rooms (identified by capital letters, in alphabetical order, from A to Z, followed by AA to AJ). Whenever a HCW visited a room with a patient with identical viral consensus sequence (i.e., same genetic profile), the field is colored according to the genetic profile (as in Figure 1A) (i.e., cases with the highest likelihood of direct transmission). HD, hemodialysis.

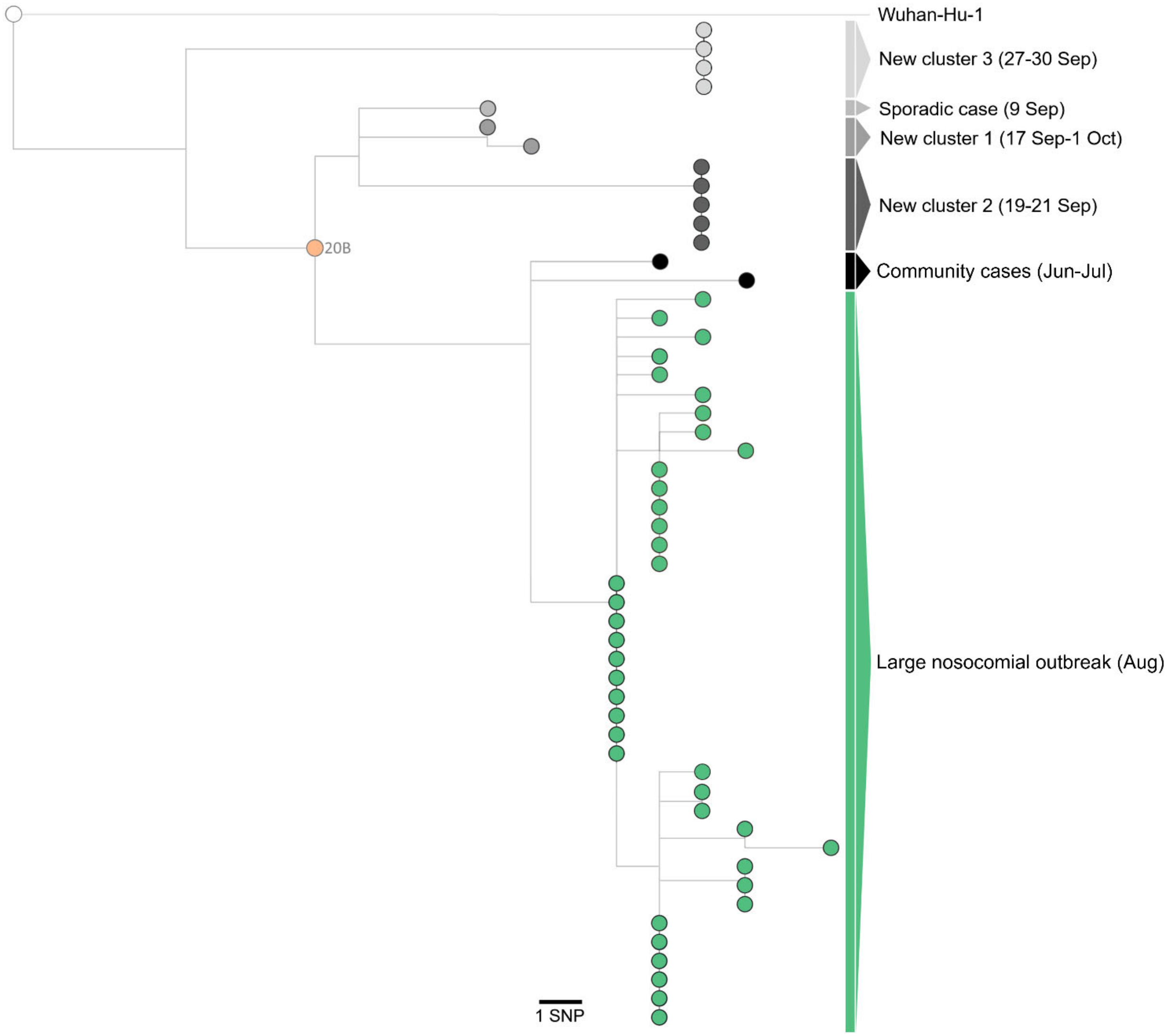

Figure S2. Independent hospital introductions of SARS-CoV-2 cases after the large nosocomial outbreak led to limited transmission chains. Maximum likelihood phylogenetic tree of SARS-CoV-2 genome sequences analysed during the investigation of SARS-CoV-2 positive cases detected in a non-COVID-19 ward of a large hospital in Portugal, between August and October 2020. The tree includes viral consensus sequences analyzed during the large nosocomial outbreak (n = 40) in August (presented in the same order as in Figure 2), as well as 11 out of 15 new positive cases detected between 9 September and 1 October. To provide better phylogenetic context, the two closest sequences of the outbreak SARS-CoV-2 variant detected in community (Portugal/PT1550/2020, collected in 21 June 2020, and Portugal/PT1614/2020, collected in 8 July 2020; https://insaflu.insa.pt/covid19/; as of 9 November 2020) and the SARS-CoV-2 reference genome sequence (used as root; Wuhan-Hu-1/2019; MN908947.3) were also included.

Supplementary Table S1. List of outbreak-related SARS-CoV-2 genome sequences generated in this study.

Sample designation	Patient / HCW code	GISAID Accession ID
Portugal/PT1701/2020	1	EPI_ISL_693630
Portugal/PT1702/2020	2	EPI_ISL_693631
Portugal/PT1703/2020	3	EPI_ISL_693632
Portugal/PT1704/2020	4	EPI_ISL_693633
Portugal/PT1705/2020	5	EPI_ISL_693634
Portugal/PT1706/2020	6	EPI_ISL_693635
Portugal/PT1707/2020	7	EPI_ISL_693523
Portugal/PT1708/2020	8	EPI_ISL_693636
Portugal/PT1709/2020	9	EPI_ISL_693520
Portugal/PT1710/2020	10	EPI_ISL_693637
Portugal/PT1711/2020	11	EPI_ISL_693638
Portugal/PT1712/2020	12	EPI_ISL_693639
Portugal/PT1713/2020	13	EPI_ISL_693640
Portugal/PT1714/2020	14	EPI_ISL_693641
Portugal/PT1715/2020	15	EPI_ISL_693642
Portugal/PT1716/2020	16	EPI_ISL_693643
Portugal/PT1717/2020	17	EPI_ISL_693644
Portugal/PT1718/2020	18	EPI_ISL_693645
Portugal/PT1719/2020	19	EPI_ISL_693646
Portugal/PT1720/2020	20	EPI_ISL_693647
Portugal/PT1721/2020	21	EPI_ISL_693648
Portugal/PT1722/2020	22	EPI_ISL_693649
Portugal/PT1723/2020	23	EPI_ISL_693519
Portugal/PT1724/2020	24	EPI_ISL_693650
Portugal/PT1725/2020	25	EPI_ISL_693651
Portugal/PT1726/2020	26	EPI_ISL_693652
Portugal/PT1727/2020	27	EPI_ISL_693653
Portugal/PT1728/2020	28	EPI_ISL_693654
Portugal/PT1729/2020	29	EPI_ISL_693518
Portugal/PT1730/2020	30	EPI_ISL_693524
Portugal/PT1731/2020	31	EPI_ISL_693655
Portugal/PT1732/2020	32	EPI_ISL_693537
Portugal/PT1733/2020	33	EPI_ISL_693656
Portugal/PT1734/2020	34	EPI_ISL_693539
Portugal/PT1735/2020	35	EPI_ISL_693534
Portugal/PT1736/2020	36	EPI_ISL_693657
Portugal/PT1737/2020	37	EPI_ISL_693536
Portugal/PT1738/2020	38	EPI_ISL_693516
Portugal/PT1739/2020	39	EPI_ISL_693535

Supplementary Table S2. Genome background of the outbreak-related SARS-CoV-2.

Genome position[a]	Nucleotide change	Amino acid change
**241**[b,c]	C>T	(5'UTR)
**1457**[c]	C>T	NSP2: R218C (non-syn) / ORF1a: R398C (non-syn)
**3037**[b,c]	C>T	NSP3: F106F (syn) / ORF1a: F924F (syn)
**6500**	C>T	NSP3: P1261S (non-syn) / ORF1a: P2079S (non-syn)
**7006**[c]	C>T	NSP3: T1429T (syn) / ORF1a: T2247T (syn)
**14408**[b,c]	C>T	NSP12b: P314L (non-syn) / ORF1b: P314L (non-syn)
**22088**[c]	C>T	S: L176F (non-syn)
**23403**[b,c]	A>G	S: D614G (non-syn)
**24019**[c]	A>G	S: E819E (syn)
**28846**[c]	C>T	N: R191R (syn)
**28881-3**[b,c]	GGG>AAC	N: R203K, G204R (non-syn)
**29692**	G>T	(3'UTR)

[a] Genome positions refer to the reference SARS-CoV-2 Wuhan-Hu-1/2019 sequence (GenBank accession MN908947).

[b] Nextstrain 20B clade SNP markers.

[c] Mutations shared with not-related community-circulating virus (Portugal/PT1550/2020 and Portugal/PT1614/2020) detected in Lisbon and Tagus Valley region in June-July 2020 (https://insaflu.insa.pt/covid19/; as of 11 December 2020)

syn: synonymous mutation.

non-syn: non-synonymous mutation.
